# Treatment-related mortality among children with cancer in Denmark during 2001–2021

**DOI:** 10.2340/1651-226X.2024.27731

**Published:** 2024-05-07

**Authors:** Marie C. L. Sørensen, Mie M. Andersen, Klaus Rostgaard, Kjeld Schmiegelow, Torben S. Mikkelsen, Peder S. Wehner, Marianne Olsen, Signe H. Søegaard, Lisa L. Hjalgrim

**Affiliations:** a Department of Paediatric Haematology and Oncology, Department of Paediatric and Adolescence Medicine, Juliane Marie Center, Copenhagen University Hospital, Copenhagen, Denmark; b Danish Cancer Institute, Danish Cancer Society, Copenhagen, Denmark; c Department of Epidemiology Research, Statens Serum Institut, Copenhagen, Denmark; d Institute for Clinical Medicine, Faculty of Medicine, University of Copenhagen, Copenhagen, Denmark; e Department of Paediatrics and Adolescent Medicine, Aarhus University Hospital, Denmark; f Department of Paediatric Haematology and Oncology, Hans Christian Andersen Children’s Hospital, Odense University Hospital, Odense, Denmark; g Department of Paediatrics and Adolescent Medicine, Section of Paediatric Haematology and Oncology, Aalborg University Hospital, Aalborg, Denmark

**Keywords:** Treatment-related death, paediatric cancer, cohort study, register based study

## Abstract

**Background:**

Survival of children with cancer has markedly improved over recent decades, largely due to intensified treatment regimes. The intensive treatment may, however, result in fatal complications. In this retrospective cohort study, we assessed temporal variation in the incidence of treatment-related death and associated risk factors among children diagnosed with cancer in Denmark during 2001–2021.

**Method:**

Among all children diagnosed with first incident cancer before age 15 years recorded in the Danish Childhood Cancer Register (*n* = 3,255), we estimated cumulative incidence of treatment-related death (death in the absence of progressive cancer) within 5 years from diagnosis using Aalen–Johansen estimators and assessed associated risk factors using Cox regression.

**Results:**

Among all 3,255 children with cancer, 93 (20% of all 459 deaths) died from treatment. Of these treatment-related deaths, 39 (42%) occurred within 3 months of diagnosis. The 5-year cumulative incidences of treatment-related death were 3.3% during 2001–2010 and 2.5% during 2011–2021 (*p* = 0.20). During 2011–2021, treatment-related deaths accounted for more than half of all deaths among children with haematological cancers. Risk factors varied according to cancer group and included female sex, age below 1 year at diagnosis, disease relapse, stem cell transplantation, central nervous system involvement, and metastasis at diagnosis.

**Interpretation:**

Despite increasing treatment intensities, the incidence of treatment-related death has remained stable during the past 20 years in Denmark. Still, clinical attention is warranted to prevent treatment-related deaths, particularly among children with haematological cancers. Patient characteristics associated with increased treatment-related death risk support patient-specific treatment approaches to avoid these fatalities.

## Introduction

The survival of children with cancer has improved markedly in recent decades primarily due to optimisation of risk stratification, intensification of therapy, and improvements in supportive care through international collaboration and clinical trials [1–3]. With access to modern health care, 5-year survival in children with cancer currently exceeds 80% in Europe and North America [1, 4–6].

The intensive treatment regimens of contemporary childhood cancer therapy is accompanied by increased risk of severe and sometimes fatal complications [7–9]. Treatment-related deaths can by definition occur at different stages of cancer therapy, that is during induction therapy, maintenance treatment, relapse therapy, and in rare cases even before initiation of anticancer treatment [10]. Indeed, studies from Canada and the Netherlands have found that up to one in four deaths among children with cancer are treatment-related [11, 12]. This underscores treatment-related death as a substantial contributor to overall childhood cancer mortality in high-income countries [10]. Importantly, the potential for preventing such deaths exists through the careful balancing of therapeutic interventions, advancements in supportive care, and the management of critical conditions in patients identified as high risk for treatment-related death [10]. Consequently, delineation of risk factors for treatment-related death is essential to help formulate preventive strategies and potentially refine targeted treatment protocols.

Due to its increasing impact on childhood cancer mortality [7, 13–17], treatment-related death has received growing attention in recent decades [7, 12–14, 17, 18]. Most studies have focused on haematological cancers [1, 14, 19–21], while the scale of treatment-related death among children with central nervous system (CNS) tumours or solid tumours has been less well-studied [8, 9]. In addition, population-based studies assessing the extent of treatment-related deaths across all childhood cancers are few [11, 12, 17]. The distinction between deaths due to progressive cancer and treatment-related deaths is important for the design of future treatment strategies in order to improve the overall cancer survival [10].

Investigations of specific causes of treatment-related death have found infection to be the most common cause followed by respiratory complications and haemorrhage [11, 12, 14, 16, 22]. However, because definitions of treatment-related death have differed between studies, and across diseases, it has proven difficult to compare and interpret investigations in different populations or in different time periods [8, 19]. Thus, it is not clear from the literature if and how the magnitude and composition of causes of death have varied over time.

To overcome this, the International Paediatric Oncology Mortality Classification Group (IPOMCG), a panel of experts on supportive care in paediatric haematology and oncology from USA, Canada, and Europe, recently developed a consensus-based definition of treatment-related death, defining it as death in the absence of progressive cancer. In addition, an algorithm to assist in the classification of death including a guideline to assess the primary cause of treatment-related death was developed [10]. We used this novel definition to assess temporal variations in the incidence of treatment-related death occurring within 5 years of diagnosis and associated risk factors in a nationwide retrospective cohort study of all children diagnosed with first incident cancer before age 15 years in Denmark during 2001–2021.

## Materials and methods

The Danish Childhood Cancer Register (DCCR) is a nationwide clinical register that records diagnosis, treatment, and vital status (death or emigration dates) for all children diagnosed with cancer in Denmark since 1985 [23], utilising vital status data from the Danish Civil Registration System [24]. Using the DCCR, we conducted a retrospective cohort study, including all children aged below 15 years recorded with a first incident cancer diagnosis between 1 January 2001 and 31 May 2021 (*n* = 3,255). For each identified child, we retrieved information on date of birth, vital status, date of cancer diagnosis (categorised by the International Classification of Childhood Cancer, third edition, ICCC-3) [25], disease burden at diagnosis, dates of stem cell transplantation, and relapse, and cause of death.

In a recent quality assurance and update of the DCCR, we reviewed medical records of patients deceased as of November 2022 to reclassify causes of death and identify unrecorded relapses. Two reviewers (MCLS, MMA), following the IPOMCG’s consensus-based algorithm [10], differentiated between treatment-related deaths and deaths due to progressive cancer. We made the exception that deaths due to secondary cancer, accidents, suicide, or congenital diseases were classified collectively as ‘death due to other causes’ as proposed by others [11, 26]. The IPOMCG algorithm assesses clinical evidence of cancer, the use or intent to use anticancer treatment, and the presence of progressive cancer. Notably, treatment-related deaths are identified across any treatment phase, including prior to the initiation of anticancer therapies. Deaths are attributed to progressive cancer based on clinician documentation in medical records or terms indicating non-curability or end-of-life care, such as ‘no curative intent, refractory disease, resistant disease, non-responsive disease, palliative care, intervention not possible, poor prognosis, end-of-life care’ [10]. Furthermore, we adhered to the IPOMCG classification to define the primary cause of treatment-related death, recording the initial complication in instances of multiple preceding complications. For example, if sepsis leading to acute respiratory distress occurred, infection was designated as the primary cause.

### Statistical analyses

To ensure consistent classification of cause of death according to IPOMCG [10], initially, we randomly selected 30 patients from the cohort to test inter-rater reliability. Cause of death was assigned by the two reviewers individually. Agreement was assessed using percentage agreement and k-statistics (<0 = no agreement, 0–0.2 = none to slight, 0.2–0.4 = fair, 0.4–0.6 = moderate, 0.6–0.8 = substantial, 0.8–1.0 = almost perfect) [27]. The two reviewers agreed in 29/30 cases (96.7%) and *k* = 0.89 (almost perfect).

Children included in the present study were followed from date of diagnosis until date of death, emigration/loss to follow-up, 5 years after diagnosis, or end of study period (2 November 2022), whichever occurred first.

Analyses were stratified by the three major ICCC-3 cancer groups, that is, haematological cancers, CNS tumours, and solid tumours, respectively (Supplementary Table 1).

We used the cumulative incidence function based on the Aalen–Johansen estimator to estimate 5-year cumulative incidences of treatment-related death and death due to progressive cancer separately. Competing risks were handled by censoring follow-up time on other causes of death than those analysed.

Due to national legislation on medical data archiving, medical records on 62 (13% of all) deceased patients were not available for review. For these patients, information on cause of death was obtained from the DCCR.

To estimate variation in the cumulative incidence of treatment-related death between different calendar periods, we compared children diagnosed with cancer during 2001–2010 and 2011–2021, respectively. The cut-off on 1 January 2011 was chosen to compare two equally sized populations. Gray´s test was used to test for differences in cumulative incidences by calendar period, diagnosis, and patient characteristics. Chi-square test was used to calculate the *p*-value for differences between the 5-year cumulative incidences of treatment-related death and death due to progressive cancer.

We estimated 5-year cumulative incidences and hazard ratios (HRs) associated with patient characteristics, including year of diagnosis, age at diagnosis, sex, relapse status, stem cell transplantation, and disease-specific characteristics (i.e., CNS-involvement for haematological cancers, and metastasis at diagnosis for solid tumours). Relapse status (yes/no) and stem cell transplantation (yes/no) were analysed as time-dependent covariates, that is, each patient initially contributed risk time as unexposed (no relapse; no stem cell transplantation) and could change exposure categories upon the date of relapse or stem cell transplantation during follow-up. HRs with 95% confidence intervals (CIs) were estimated using Cox proportional hazards models with time since diagnosis as the underlying time scale and statistical significance was based on likelihood ratio test.

HRs were adjusted for age at diagnosis (linearly), calendar year (linearly), and sex. HRs associated with stem cell transplantation and relapse were further adjusted for relapse (time-dependent, yes/no) and stem cell transplantation (time-dependent, yes/no) respectively.

We also estimated the crude rate and cumulative incidences of treatment-related death according to time since diagnosis (0–1 month, 2–3 months, 4–5 months, and 6+ months).

In supplementary analyses, we assessed 5-year cumulative incidence of all-cause mortality, including treatment-related death, progressive cancer death, death due to other and unknown causes, and estimated HRs associated with patient characteristics.

Smoothening of cumulative incidence functions was carried out using the LOESS function. All statistical analyses were performed using R version 4.1.2 (R Project for Statistical Computing).

## Results

In total, 3,255 children aged below 15 years were registered with a diagnosis of a first incident cancer in Denmark in the period 2001–2021. Table 1 presents patient characteristics of the included children overall and according to cancer group.

**Table 1:** Characteristics of children aged below 15 years diagnosed with cancer during 2001–2021, according to cancer group.

Patient characteristics	Any childhood cancer		Haematological cancers		CNS tumours		Solid tumours	
	*n*	%	*n*	%	*n*	%	*n*	%
Total	3,255	100	1,295	100	867	100	1,093	100
Sex								
Female	1,528	47	544	42	431	50	553	51
Male	1,727	53	751	58	436	50	540	49
Age^a^	5.5	2.5, 10.5	5.3	3.0, 10.2	6.9	3.2, 10.6	4.0	1.2, 10.6
Age group								
Below 1 year	377	12	60	5	71	8	246	23
1–5 years	1,352	42	654	50	320	37	378	35
6–10 years	792	24	303	23	271	31	218	20
11–14 years	734	23	278	21	205	24	251	23
Relapse								
No	2,641	81	1,137	88	620	72	884	81
Yes	614	19	158	12	247	28	209	19
Stem cell transplant								
No	2,985	92	1,123	87	855	99	1,007	92
Yes	270	8.3	172	13	12	1	86	8
- Allogenic	162	60	161	94	0	0	1	1
- Autologous	102	38	6	3	11	92	85	99
- Unknown	6	2	5	3	1	8	0	0

a Median (interquartile range).

During follow-up, 459 children with cancer died within 5 years from diagnosis; 348 (76%) due to progressive cancer; 93 (20%) due to treatment-related causes; 9 (2%) from other causes; and 9 (2%) from unknown causes owing to lack of information on cause of death. The latter two were censored upon death in analyses of treatment-related death and death due to progressive cancer. Children with haematological cancers accounted for 70% (*n* = 65) of all treatment-related deaths (primarily acute lymphoblastic leukaemia (ALL), *n* = 40), CNS tumours, and solid tumours accounted for 17% (*n* = 16) and 13% (*n* = 12), respectively.

### Mortality among all children with cancer

**Figure 1:**
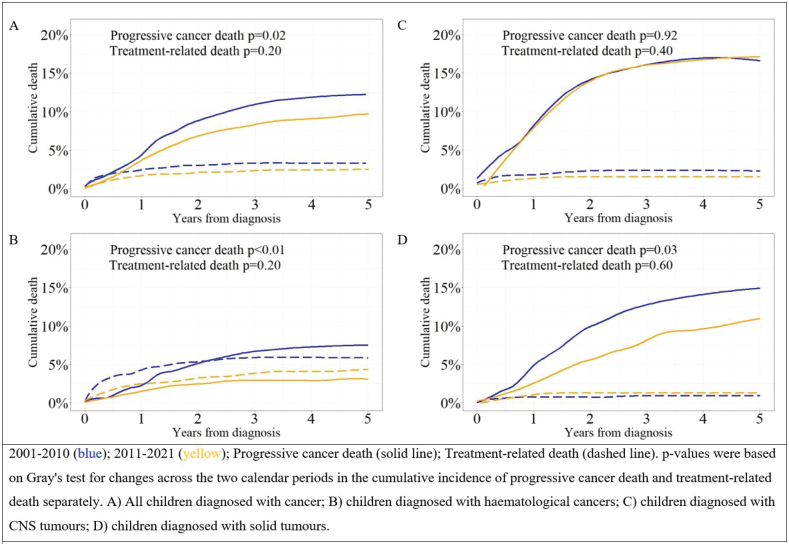
Cumulative incidence of treatment-related death and death due to progressive cancer by calendar period of diagnosis, overall and by cancer group among children diagnosed with cancer during 2001–2021.

Among all children with cancer, the 5-year cumulative all-cause mortality decreased from 16.0% during 2001–2010 to 12.0% during 2011–2021 (*p* < 0.01) (Supplementary Table 2). During the study period, the 5-year cumulative incidence of treatment-related death remained stable at 3.3% during 2001–2010 and 2.5% during 2011–2021 (*p* = 0.20). Meanwhile death due to progressive cancer decreased from 12.0% during 2001–2010 to 9.7% during 2011–2021 (*p* = 0.02) (Figure 1A). Cumulative incidence of death due to other causes was 0.3% and death without known cause was 0.3% during 2001–2021.

Of all treatment-related deaths, 23% (*n* = 21) occurred within the first month, 42% (*n* = 39) within the first 3 months, and 50% (*n* = 47) within the first 5 months after diagnosis. A higher rate of treatment-related death in proximity to diagnosis was observed across all three cancer groups, with the highest rates observed in the first month after diagnosis (Table 2).

**Table 2:** Crude rate and cumulative incidence of treatment-related death according to time since diagnosis and cancer group during 2001–2021.

Time after diagnosis	Numbers at risk (*n*)	Events (*n*)	Person-years^a^	Crude rate per 1,000^b^	∆Cumulative incidence (%)^c^
Haematological cancers					
0–1 month	1,295	10	110.7	90	0.77 (0.40, 1.40)
2–3 month	1,279	15	213.5	70	1.20 (0.69, 1.90)
4–5 month	1,261	6	211.6	28	0.48 (0.20, 1.00)
6+ month	1,253	34	5092.0	7	2.80 (2.00, 3.90)
CNS tumours					
0–1 month	867	9	73.6	122	1.00 (0.52, 1.90)
2–3 month	848	1	142.0	7	0.12 (0.01, 0.65)
4–5 month	839	1	141.0	7	0.12 (0.01, 0.66)
6+ month	830	5	3082.0	2	0.64 (0.25, 1.40)
Solid tumours					
0–1 month	1,093	2	93.8	21	0.18 (0.04, 0.64)
2–3 month	1,086	2	182.0	11	0.18 (0.04, 0.64)
4–5 month	1,081	1	181.0	6	0.09 (0.01, 0.51)
6+ month	1,072	7	4165.0	2	0.68 (0.31, 1.30)

a Person-years;

b Crude Rate (n event/1,000 person-years);

c ∆Cumulative incidence within specific time points (%) with 95% confidence intervals. CNS: central nervous system.

Patient characteristics associated with all-cause mortality in all children diagnosed with cancer are presented in Supplementary Table 2.

### Mortality among children with haematological cancers

Among children with haematological cancers, the 5-year cumulative incidence of treatment-related death was 5.9% during 2001–2010 and 4.3% during 2011–2021 (*p* = 0.20) (Table 3, Figure 1B). Meanwhile, the 5-year cumulative incidence of death due to progressive cancer decreased from 7.5% during 2001–2010 to 3.1% during 2011–2021 (*p* < 0.01) (Figure 1B). Thus, treatment-related deaths (57% of all deaths during the period) were slightly more common than deaths due to progressive cancer during 2011–2021, although the two 5-year cumulative incidences did not differ statistically significantly (*p* = 0.27, Figure 1B).

**Table 3:** Five-year cumulative incidence and relative risk (hazard ratios) of treatment-related death according to patient characteristics and cancer group among children diagnosed with cancer during 2001–2021.

Patient characteristics	Events (*n*)	Person- years	5-year Cumulative incidence (%)^a^	*p* ^b^	Crude		Adjusted	
					Hazard ratio (95% CI)	*p* ^c^	Hazard ratio (95% CI)	*p* ^c^
Haematological cancers								
Diagnostic period^d^				0.20		0.20		0.20
2001–2010	39	2,973	5.9 (4.2, 7.8)		Reference		Reference	
2011–2021	26	2,649	4.3 (2.9, 6.1)		0.70 (0.43, 1.15)		0.70 (0.43, 1.15)	
Linear trend per year	65	5,622			0.98 (0.94, 1.03)	0.50	0.99 (0.94, 1.03)	0.50
Sex^e^				< 0.01		< 0.01		0.01
Female	38	2,346	7.1 (5.1, 9.4)		Reference		Reference	
Male	27	3,277	3.6 (2.4, 5.1)		0.50 (0.31, 0.83)		0.52 (0.32, 0.85)	
Age groups^f^				< 0.01		0.01		0.02
Below 1 year	8	219	13.0 (6.2, 23.0)		2.96 (1.36, 6.43)		2.72 (1.25, 5.92)	
1–5 years	32	2,849	4.9 (3.4, 6.8)		Reference		Reference	
6–10 years	18	1,338	6.1 (3.7, 9.2)		1.20 (0.67, 2.13)		1.26 (0.71, 2.24)	
11–14 years	7	1,216	2.5 (1.1, 4.9)		0.51 (0.23, 1.16)		0.52 (0.23, 1.17)	
Relapse^g^				< 0.01		< 0.01		< 0.01
No	49	5,348	4.3 (3.2, 5.6)		Reference		Reference	
Yes	16	274	10.0 (6.1, 16.0)		22.9 (11.60, 45.10)		21.20 (9.55, 47.20)	
Stem cell transplant^h^				0.11		< 0.01		0.07
No	53	5,103	4.7 (3.5, 6.0)		Reference		Reference	
Yes	12	519	7.7 (4.3, 12.0)		5.66 (2.82, 11.40)		2.22 (0.95, 5.18)	
CNS involvement^i^				0.02		0.04		0.05
No	57	5,325	4.7 (3.6, 6.0)		Reference		Reference	
Yes	8	297	11.0 (4.9, 19.0)		2.36 (1.13, 4.95)		2.28 (1.06, 4.90)	
CNS tumours								
Diagnostic period^d^				0.40		0.40		0.30
2001–2010	9	1,666	2.3 (1.1, 4.1)		Reference		Reference	
2011–2021	7	1,768	1.5 (0.7, 2.9)		0.64 (0.24, 1.73)		0.62 (0.23, 1.66)	
Linear trend per year	16	3,434			0.96 (0.88, 1.04)	0.30	0.96 (0.88, 1.04)	0.30
Sex^e^				0.12		0.12		0.11
Female	11	1,686	2.6 (1.4, 4.4)		Reference		Reference	
Male	5	1,748	1.1 (0.4, 2.5)		0.44 (0.15, 1.27)		0.43 (0.15, 1.25)	
Age groups^f^				0.04		0.06		0.06
Below 1 year	4	250	5.6 (1.8, 13.0)		2.74 (0.80, 9.36)		2.79 (0.82, 9.55)	
1–5 years	7	1,268	2.2 (1.0, 4.3)		Reference		Reference	
6–10 years	4	1,087	1.5 (0.5, 3.5)		0.67 (0.20, 2.28)		0.67 (0.20, 2.31)	
11–14 years	1	829	0.5 (0.1, 2.5)		0.22 (0.03, 1.77)		0.21 (0.03, 1.71)	
Solid tumours								
Diagnostic period^d^				0.60		0.60		0.60
2001–2010	5	2,399	0.9 (0.4, 2.0)		Reference		Reference	
2011–2021	7	2,219	1.3 (0.6, 2.5)		1.39 (0.44, 4.39)		1.39 (0.44, 4.39)	
Linear trend per year	12	4,618			1.05 (0.95, 1.16)	0.30	1.05 (0.95, 1.16)	0.30
Sex^e^				0.50		0.50		0.50
Female	5	2,327	0.9 (0.6, 2.0)		Reference		Reference	
Male	7	2,291	1.3 (0.6, 2.6)		1.44 (0.46, 4.55)		1.44 (0.45, 4.57)	
Age groups^f^				0.30		0.13		0.14
Below 1 year	3	1,061	1.2 (0.4, 3.3)		0.78 (0.20, 3.13)		0.75 (0.19, 3.01)	
1–5 years	6	1,579	1.6 (0.7, 3.3)		Reference		Reference	
6–10 years	0	932	–		–		–	
11–14 years	3	1,047	1.2 (0.3, 3.2)		0.76 (0.19, 3.04)		0.79 (0.20, 3.17)	
Stem cell transplant^h^				< 0.01		< 0.01		< 0.01
No	7	4,347	0.7 (0.3, 1.4)		Reference		Reference	
Yes	5	270	5.8 (2.1, 12.0)		41.80 (8.04, 217.00)		46.90 (8.60, 256.00)	
Metastasis^g^				0.01		0.01		0.02
No	5	3,598	0.6 (0.2, 1.4)		Reference		Reference	
Yes	7	1,019	2.5 (1.1, 4.9)		4.22 (1.34, 13.30)		4.09 (1.30, 12.90)	

All analyses were implicitly adjusted for time since diagnosis (underlying timescale).

a 5-year cumulative incidence (%) with 95% confidence intervals

b Gray’s Test

c Likelihood ratio test

d Adjusted for age at diagnosis and sex

e Adjusted for year of diagnosis and age at diagnosis

f Adjusted for year of diagnosis and sex

g Adjusted for year of diagnosis, age at diagnosis, sex and stem cell transplant (time-dependent)

h Adjusted for year of diagnosis, age at diagnosis, sex and relapse (time-dependent)

i Adjusted for year of diagnosis, age at diagnosis and sex. CNS: central nervous system.

While the rate of treatment-related death was highest within the first 3 months after diagnosis, 52% (*n* = 34) of all treatment-related deaths occurred 6 months or more after diagnosis (Table 2). Among these, 65% (*n* = 22) had experienced a relapse (*n* = 10), received a stem cell transplantation (*n* = 6) or both (*n* = 6).

The 5-year cumulative incidence of treatment-related death was higher among girls (7.1%) than among boys (3.6%, *p* < 0.01), corresponding to an adjusted HR of 0.52 (95% CI: 0.32–0.85) for boys compared with girls (Table 3). This sex-difference was evident in both calendar periods (i.e., 2001–2010 and 2011–2021). In contrast, 5-year cumulative incidence of death due to progressive cancer was higher among boys (6.5%) compared with girls (3.9%, *p* = 0.05) during 2001–2021.

Further, children aged less than 1 year at diagnosis had the highest 5-year cumulative incidence of treatment-related death (13.0%) compared with other age groups (*p* < 0.01). Among children aged less than 1 year, 50% were diagnosed with ALL, 25% with acute myeloid leukaemia (AML), and 25% with unspecified leukaemia. Compared with children aged 1–5 years at diagnosis, children aged less than 1 year had a 2.72-fold (95% CI: 1.25, 5.92) increased relative risk of treatment-related death (Table 3).

Children experiencing a relapse had a 21.20-fold (95% CI: 9.55, 47.20) increased relative risk of treatment-related death compared with children who did not experience a relapse. Stem cell transplantations – all of which were allogenic – were associated with a 5.66-fold (95% CI: 2.82, 11.40) increased relative risk of treatment-related death in the crude model. However, this association was not statistically significant when adjusting for other patient characteristics (HR = 2.22 (95% CI: 0.95, 5.18) (Table 3). Among children treated with stem cell transplantation, 78 also experienced a relapse (*n* = 68 prior to transplantation and *n* = 10 after stem cell transplantation).

In total, 6% (*n* = 77) of children with haematological cancers had CNS involvement at diagnosis, and among these, the relative risk of treatment-related death was 2.28-fold (95% CI: 1.06, 4.90) increased compared with children without CNS involvement (Table 3).

### Mortality among children with CNS tumours

Among children with CNS tumours, the 5-year cumulative incidence of treatment-related death was 2.3% during 2001–2010 and 1.5% during 2011–2021 (*p* = 0.40) (Figure 1C and Table 3). Death due to progressive cancer was the most frequent cause of death with a 5-year cumulative incidence of 17.0% during both calendar periods (*p* = 0.92) (Figure 1C).

Most treatment-related deaths (56%, *n* = 9) occurred within the first month after diagnosis with a cumulative incidence of 1.0% (Table 2).

Girls tended to have a higher risk of treatment-related death than boys with 5-year cumulative incidences of 2.6% and 1.1%, respectively (*p* = 0.12) (Table 3). Children aged less than 1 year at diagnosis had the highest 5-year cumulative incidence of treatment-related death (5.6%) compared with older ages at diagnosis (*p* = 0.04, Table 3).

The risks of treatment-related death among children with relapse and children with stem cell transplantation were not estimated due to few events.

### Mortality among children with solid tumours

The 5-year cumulative incidence of treatment-related death was stable during the study period: 0.9% during 2001–2010 and 1.3% during 2011–2021 (*p* = 0.60) (Figure 1D and Table 3). In contrast, the 5-year cumulative incidence of death due to progressive cancer decreased from 15.0% during 2001–2010 to 11.0% during 2011–2021 (*p* = 0.03) (Figure 1D).

The few treatment-related deaths among children with solid tumours (*n* = 12 during 2001–2021) limited the ability to detect possible risk factors. In total, 26% (*n* = 282) of children with solid tumours had metastases at diagnosis, and among these seven children died from treatment-related complications. This corresponded to a 4.09-fold (95% CI: 1.30, 12.90) increased relative risk compared with children without metastases at diagnosis (Table 3). Moreover, children treated with stem cell transplantation had a 5-year cumulative incidence of treatment-related death of 5.8% compared with 0.7% for children who did not receive such treatment (based on 5 and 7 treatment-related deaths, respectively, *p* < 0.01, Table 3). The risks of treatment-related death among children with relapse were not estimated due to few events.

### Primary cause of treatment-related deaths

Overall, the three most common primary causes of treatment-related deaths were: (1) infection (37%, *n* = 34 with or without documented microbiology), (2) nervous system complications (14%, *n* = 13), including necrosis, encephalopathy, stroke, hydrocephalus with raised intercranial pressure, and (3) haemorrhage (11%, *n* = 10), including intercranial and pulmonary haemorrhage (Table 4).

**Table 4:** Primary cause of treatment-related deaths among all children with cancer and according to cancer group during 2001–2021.

Primary cause of treatment related death.	Any childhood cancer		Haematological cancers		CNS tumours		Solid tumours	
	*n*	%	*n*	%	*n*	%	*n*	%
Total	93	100	65	100	16	100	12	100
Infection	34	37	27	42	2	12	5	42
Nervous system	13	14	5	8	7	44	1	8
Haemorrhage	10	11	4	6	3	19	3	25
Respiratory system	8	9	6	9	0	0	2	17
Gastrointestinal system	5	5	5	8	0	0	0	0
Immune mediated	4	4	4	6	0	0	0	0
Cardiac system	3	3	1	2	1	6	1	8
Thrombosis	3	3	3	5	0	0	0	0
Metabolic	1	1	1	2	0	0	0	0
Renal	1	1	1	2	0	0	0	0
Unknown	11	12	8	12	3	19	0	0

CNS: central nervous system.

Among children with haematological cancers, infection was the most common primary cause accounting for 42% of treatment-related deaths (39% among girls and 44% among boys). Infections were also the most common cause of treatment-related death among children with solid tumours (42%) and among children with relapse and/or stem cell transplantation (results not shown). Among children with CNS tumours, nervous system complications (44%) were the most common cause of treatment-related death (Table 4).

Within the first month of diagnosis, nervous system complications were the most frequent cause of treatment-related death (39%, *n* = 7 distributed among children with haematological cancers and CNS tumours), whereas haemorrhage (28%, *n* = 5) was the second most frequent cause with cases observed among all three cancer groups.

## Discussion

Using a consensus-based international definition of treatment-related death, we investigated temporal variations in the cumulative incidence among all children diagnosed with cancer in Denmark between 2001 and 2021. Overall, we found that one in five deaths among children with cancer were treatment-related, and that 42% of these occurred within the first 3 months after diagnosis. This aligns with findings from the few other population-based investigations of heterogeneous childhood cancer populations using the IPOMCG classification of treatment-related death [11, 12, 17]. Across all childhood cancers, the incidence of treatment-related death did not vary notably in Denmark from 2001 to 2021. Treatment-related deaths accounted for approximately half of all deaths among children with haematological cancers, but were rare among children with CNS and solid tumours.

Successful treatment of children with cancer entails finding the balance between giving sufficient treatment to achieve remission and minimising the risk of severe complications of treatment. The stable incidence of treatment-related deaths observed during the past 20 years likely reflects an interplay of several factors. Our findings align with the notion that enhanced supportive care has counterbalanced the complication risks associated with more intensive treatment regimens, notably in haematological cancers where survival advances specifically have been achieved by intensified treatment in both first and second-line therapies [4, 18, 28]. Moreover, advances in treatment protocols, informed by refined risk stratification that incorporate genetic profiling, assessments of minimal residual disease, targeted therapies, and improved surgery have also played a substantial role in stabilising treatment-related death risk and reducing deaths due to progressive cancer as observed among children with haematological cancers and solid tumours [4, 29–31]. Of note, the observation that treatment-related deaths accounted for half of all deaths in children with haematological cancers in recent years reflected a greater prevention of deaths due to progressive cancer during the same period.

In the present study, we confirmed previously reported risk factors for treatment-related death in children with haematological cancers and solid tumours, notably, relapse and stem cell transplantation [11, 12, 17]. The necessity for additional intensified chemotherapy cycles, in combination with the immunosuppression characteristic of patients who relapse and undergo stem cell transplantation, likely explain the increased risk of dying from treatment [22]. Specifically, we observed that relapse and stem cell transplantation were major contributors to the treatment-related deaths occurring 6 months or more following diagnosis in children with haematological cancers.

Furthermore, in line with previous observations [11, 17], we found that children aged less than 1 year were at the highest risk of dying from treatment compared with older children with haematological cancers and CNS tumours. This increased risk likely reflects more aggressive disease presentation, greater fragility, complication susceptibility, and difficulties in symptom interpretation in this age group [15, 32, 33].

We observed higher cumulative incidences of treatment-related death among girls compared with boys with haematological cancers and CNS tumours. Results of previous studies align with this observation [11, 12, 34]. While a Nordic study of children with ALL found that girls more often died from treatment-related infections than boys [13], our findings did not support this notion. Others have shown that girls with ALL generally suffer more treatment-related toxicities than boys [35], yet the mechanisms underlying these sex differences remain elusive and warrant further investigation.

To improve supportive care and to guide the design of pre-emptive interventions, future studies should encompass auditing and detailed, systematic recording of patient courses. This approach will provide further insights into which patient groups are particularly vulnerable to dying from treatment and require treatment adaptions.

We observed that the rates of treatment-related deaths were highest in the first months after diagnosis. In addition, children with haematological cancers or solid tumours were particularly vulnerable to dying from infections. For children with haematological cancers in particular, this vulnerability likely resulted from the combined immunodeficiency induced by the disease itself and the intensive treatment with chemotherapy and steroids, which is most pronounced in the early treatment phase [7, 21, 34]. Moreover, children with CNS tumours who died from treatment frequently succumbed to nervous system complications, often within the first month of diagnosis, suggesting that these deaths occurred in connection with primary tumour presentation and surgery. Overall, the distribution of causes of treatment-related deaths observed in the present study is congruent with results of previous investigations [11, 12, 17, 34]. Of note, however, the classification of primary cause of treatment-related death is challenged by the multiple complications experienced by the child that often precede these deaths, and likely cause discrepancy between studies.

This study has several strengths. The population-based design allowed us to assess the cumulative incidence of treatment-related death among all children with cancer in Denmark during the past 20 years, across different treatment trials and protocols. In addition, the DCCR has real time registration of all incident cases of childhood cancer and their vital status, ensuring updated and close to complete follow-up. Finally, we used a consensus-based definition of treatment-related death, which strengthens the comparison of different treatment approaches over time as well as with other settings. Treatment of children with cancer in Denmark adheres to Nordic and European protocols, and the universal healthcare system ensures that children from all socio-economic groups and geographic regions are treated. This renders our study’s findings broadly applicable to other high-income countries.

The study also has limitations. First, despite utilising a consensus-based definition, the intricate task of differentiating treatment-related deaths from those due to progressive cancer, especially in the early treatment phases, may have led to some misclassification. Second, our validation of the DCCR was constrained to identifying unrecorded relapses only among deceased patients, possibly leading to an overestimation of treatment-related death risk in children with relapsed cancer. Moreover, despite the population-based nature of this study, including more than 3,000 children with cancer, the rarity of treatment-related deaths limited the statistical precision of risk estimates and the ability to detect risk factors.

In conclusion, while treatment intensities have increased substantially in the last 20 years, the incidence of treatment-related death has remained constant among children with cancer in Denmark. Still, treatment-related death warrants further clinical attention as one in five deaths among children with cancer overall are treatment-related, representing half of all deaths among children with haematological cancers. The risk of treatment-related death varied according to specific patient characteristics, which suggests a role for patient specific treatment approaches.

## Supplementary Material

Treatment-related mortality among children with cancer in Denmark during 2001–2021

## Data Availability

The data that support the findings of this study are available from the Danish Clinical Quality Program–National Clinical Registries (RKKP). Restrictions apply to the availability of these data, which were used under license for this study. Data are available with the permission of RKKP.
