# Supplementary material for: Treatment-related mortality among children with cancer in Denmark during 2001–2021
Source: Acta Oncol. 2024 May 7;63:27731. doi: 10.2340/1651-226X.2024.27731 (PMC11332509; doi:10.2340/1651-226X.2024.27731)
Supplement: Treatment-related mortality among children with cancer in Denmark during 2001–2021 [file AO-63-27731-s1.pdf]

Supplementary material has been published as submitted. It has not been copyedited, or typeset by Acta Oncologica

Supplementary Table 1. Cancer groups according to International Classification of Childhood Cancer, Third Edition (ICCC-3)

Haematological cancers	n
I. Leukaemias, myeloproliferative and myelodysplastic diseases	984
II. Lymphomas and reticuloendothelial neoplasms	311
**Solid tumours**	**n**
IV. Neuroblastoma and other peripheral nervous cell tumours	166
V. Retinoblastoma	96
VI. Renal tumours	146
VII. Hepatic tumours	48
VIII. Malignant Bone tumours	153
IX. Soft tissue and other extraosseous sarcomas	186
Xb-e. Germ cell tumours, trophoblastic tumours and neoplasms of gonads	176
XI. Other malignant epithelial neoplasms and malignant melanomas	103
XII. Other and unspecified malignant neoplasms[a]	18
**CNS tumours**	**n**
III. CNS and miscellaneous intracranial and intraspinal neoplasms	855
Xa. Germ cell tumours, trophoblastic tumours and neoplasms of gonads	9
XII. Other and unspecified malignant neoplasms[a]	2
[a]Tumour type registered, but specific ICCC3 diagnosis not available.	

Supplementary Table 2. 5-year cumulative incidence and relative risk (hazard ratios) of all-cause mortality according to patient characteristics among children diagnosed with cancer during 2001–2021.

	Events n	Person years	5-year Cumulative incidence (%)[a]	p[b]	Crude Hazard ratio (95%CI)	Crude p[c]	Adjusted Hazard ratio (95%CI)	Adjusted p[c]
Diagnostic period[d]				<0.01		<0.01		<0.01
2001-2010	264	7038	16.0 (15.0, 18.0)		Reference		Reference	
2011-2021	195	6636	12.0 (11.0, 14.0)		0.73 (0.61, 0.88)		0.73 (0.61, 0.88)	
Linear trend per year					0.97 (0.95, 0.98)	<0.01	0.97 (0.95, 0.98)	<0.01
Sex[e]				0.40		0.40		0.40
Female	222	6358	15.0 (13.0, 17.0)		Reference		Reference	
Male	237	7316	14.0 (12.0, 16.0)		0.93 (0.77, 1.12)		0.93 (0.77, 1.12)	
Age groups[f]				0.80		0.80		0.80
Below 1 year	57	1530	15.0 (12.0, 19.0)		1.15 (0.86, 1.55)		1.18 (0.87, 1.58)	
1-5 years	184	5695	14.0 (12.0, 16.0)		Reference		Reference	
6-10 years	112	3357	14.0 (12.0, 17.0)		1.03 (0.82, 1.31)		1.04 (0.82, 1.31)	
11-15 years	106	3092	15.0 (12.0, 18.0)		1.06 (0.83, 1.34)		1.05 (0.83, 1.34)	
Diagnosis[g]				<0.01		<0.01		<0.01
CNS tumour	165	3434	19.0 (17.0, 22.0)		Reference		Reference	
Haematological cancer	140	5622	11.0 (9.4, 13.0)		0.53 (0.43, 0.67)		0.53 (0.42, 0.66)	
Solid tumour	154	4618	15.0 (13.0, 17.0)		0.70 (0.57, 0.88)		0.70 (0.56, 0.87)	
Relapse[h]				<0.01		<0.01		<0.01
No	177	12469	6.8 (5.8, 7.8)		Reference		Reference	

Yes	282	1205	47.0 (43.0, 51.0)		35.2 (28.3, 43.8)		36.2 (28.9, 45.3)	
Stem cell transplant[i]				<0.01		<0.01		<0.01
No	388	12855	13.0 (12.0, 14.0)		Reference		Reference	
Yes	71	819	28.0 (22.0, 33.0)		3.80 (2.93, 4.93)		2.24 (1.71, 2.94)	

All analyses were implicitly adjusted for time since diagnosis (underlying timescale).

[a] 5-year cumulative incidence (%) with 95% confidence intervals; [b] Gray's test; [c] Likelihood ratio test

[d] Adjusted for age at diagnosis and sex; [e] Adjusted for year of diagnosis and age at diagnosis; [f] Adjusted for year of diagnosis and sex; [g] Adjusted for year of diagnosis, age at diagnosis and sex; [h] Adjusted for year of diagnosis, age at diagnosis, sex and stem cell transplant (time-dependent); [h] Adjusted for year of diagnosis, age at diagnosis, sex and stem cell transplant (time-dependent); [i] Adjusted for year of diagnosis, age at diagnosis, sex, and relapse (time-dependent).
